# Living with sickle cell in Uganda: A comprehensive perspective on challenges, coping strategies, and health interventions

**DOI:** 10.1097/MD.0000000000041062

**Published:** 2024-12-20

**Authors:** Emmanuel Ifeanyi Obeagu, Getrude Uzoma Obeagu

**Affiliations:** a Department of Medical Laboratory Science, Kampala International University, Ishaka, Uganda; b School of Nursing Science, Kampala International University, Ishaka, Uganda.

**Keywords:** challenges, coping strategies, genetic counseling, living with sickle cell, quality of life, sickle cell anemia, Uganda

## Abstract

Sickle cell anemia (SCA) is a hereditary blood disorder with profound implications for affected individuals, particularly in resource-limited settings such as Uganda. This review explores the multifaceted aspects of SCA in Uganda, focusing on epidemiology, challenges faced by individuals, coping strategies, healthcare disparities, and community support. The study incorporates a thorough examination of the genetic landscape, prevalence, and the impact of SCA on the quality of life in Uganda. Coping strategies and resilience play a pivotal role in mitigating the impact of SCA on affected individuals. This review critically evaluates the various coping mechanisms employed by individuals in Uganda and the resilience demonstrated in the face of chronic illness. It explores the psychological, social, and cultural dimensions of coping and resilience, shedding light on adaptive strategies that contribute to improved quality of life. This article aims to contribute valuable insights into the specific challenges faced by individuals with SCA in Uganda, offering a foundation for targeted interventions, improved healthcare policies, and increased awareness within both the medical community and the broader society.

## 1. Introduction

Sickle cell disease (SCD) is a hereditary blood disorder characterized by the production of abnormal hemoglobin, which leads to the formation of sickle-shaped red blood cells. These distorted cells can cause blockages in blood vessels, leading to pain, organ damage, and increased susceptibility to infections. SCD is a global health concern, but its impact is particularly profound in sub-Saharan Africa, where genetic factors, high birth rates, and limited healthcare resources contribute to its high prevalence. Uganda, located in the heart of this region, is one of the countries with a significant burden of SCD. The disease affects ≈1% to 2% of the Ugandan population, with an estimated 150,000 individuals living with the condition.^[1]^ The genetic basis of SCD stems from a mutation in the hemoglobin gene, leading to the production of hemoglobin S (HbS). Individuals who inherit 2 copies of the HbS gene (one from each parent) develop SCD, while those with only 1 copy are carriers of the sickle cell trait. In Uganda, the high carrier rate contributes to the persistence of SCD across generations. Despite the high prevalence, there is often a lack of awareness about the disease, which can delay diagnosis and treatment.^[2]^ Healthcare access for individuals with SCD in Uganda is a major challenge. Many patients, especially those in rural areas, face difficulties reaching medical facilities due to poor infrastructure and limited transportation options. Urban centers, where most healthcare facilities are located, are often overcrowded and underresourced, further complicating access to timely and effective medical care. In addition, the costs associated with managing SCD, including medical consultations, medications, and hospitalizations, pose a significant financial burden on families. This economic strain can limit patients’ ability to access the necessary treatments and follow-up care.^[3]^ The quality of healthcare services available to patients with SCD in Uganda is often inconsistent. While some healthcare facilities are equipped with the necessary diagnostic tools and treatments for SCD, many lack essential resources such as trained personnel, reliable diagnostic equipment, and adequate medication supplies. This disparity in healthcare quality affects the effectiveness of treatment and the management of SCD complications. Addressing these issues requires a comprehensive approach to strengthening healthcare infrastructure and ensuring that all patients receive high-quality care.^[4]^

Social stigma and discrimination are significant issues faced by individuals with SCD in Uganda. Misconceptions and a lack of understanding about the disease contribute to the stigmatization of affected individuals, which can lead to social exclusion and emotional distress. Public awareness campaigns and educational initiatives are essential for dispelling myths about SCD and fostering a more supportive environment for patients and their families. Reducing stigma is a key step in improving the quality of life for individuals with SCD.^[5]^ The psychosocial impact of SCD extends beyond the physical symptoms of the disease. Individuals with SCD and their families often experience emotional challenges, including anxiety and depression, due to the chronic nature of the illness and the associated social and financial difficulties. Support systems, including counseling services and peer support groups, play a vital role in helping patients cope with the emotional burden of SCD. Understanding these psychosocial aspects is crucial for developing comprehensive care strategies that address both physical and mental health needs.^[6]^ Coping strategies employed by individuals with SCD include a variety of self-management techniques and support mechanisms. Patients often rely on pain management strategies, such as medication and home remedies, to cope with pain crises. In addition, family support and community networks provide essential emotional and practical assistance. These coping strategies are a testament to the resilience of individuals living with SCD, but they also highlight the need for more robust and systematic support systems.^[7]^ Current health interventions in Uganda for managing SCD include public health programs focused on early diagnosis, treatment, and prevention. Newborn screening programs are designed to identify SCD cases early, allowing for timely intervention and management. Genetic counseling services aim to educate prospective parents about the risks of SCD and offer guidance on family planning. These interventions are crucial for improving outcomes for individuals with SCD, but there is a need for increased awareness and expansion of these services to reach more people.^[8]^ Policy development and implementation are critical for addressing the challenges associated with SCD in Uganda. Effective policies should focus on improving healthcare access, reducing financial barriers, and addressing social stigma. Collaborations between government agencies, nongovernmental organizations (NGOs), and international bodies are essential for the successful implementation of these policies. Advocating for comprehensive health policies and fostering partnerships are key strategies for advancing SCD care and support in Uganda.^[8]^

## 2. Aim

The primary aim of this review article is to provide a comprehensive examination of SCD in Uganda, focusing on the multifaceted challenges faced by individuals living with the condition, the coping strategies employed, and the effectiveness of existing health interventions.

## 3. Rationale

The rationale for this review article is grounded in the recognition of SCD as a significant public health challenge in Uganda, characterized by a high prevalence rate and profound impacts on affected individuals and communities. Uganda has one of the highest prevalence rates of SCD in the world, with significant public health implications. According to recent estimates, ≈1 in 20 Ugandans are carriers of the sickle cell trait, and the prevalence of SCD among children is also high. Despite this, there is a need for a more detailed and updated understanding of the epidemiological trends, which this review aims to address. By consolidating and analyzing current data on SCD prevalence and incidence, the review aims to provide a clearer picture of the disease’s burden in Uganda. Individuals living with SCD in Uganda face a range of complex challenges, including inadequate access to healthcare, high treatment costs, and insufficient support systems. These challenges are compounded by socioeconomic factors and limited healthcare infrastructure. There is a need for a thorough examination of these challenges to identify gaps in the current healthcare system and develop effective solutions. This review aims to gather and synthesize information on these challenges to inform policy recommendations and improve patient care. While there is some existing research on coping strategies, a comprehensive review that aggregates various approaches and evaluates their effectiveness is lacking. This review seeks to fill this gap by assessing the coping mechanisms employed by patients with SCD and caregivers, thereby providing insights into effective support strategies. Health interventions for SCD in Uganda have evolved over time, but there is a need to systematically evaluate their effectiveness and impact. This review aims to analyze various health interventions, including newborn screening programs, treatment protocols, and public health initiatives, to determine their success and identify areas for improvement. Evaluating these interventions is crucial for optimizing SCD management strategies and informing future public health policies. Current literature often focuses on specific aspects of SCD, such as clinical outcomes or individual interventions, without integrating these elements into a holistic understanding of SCD management. This review aims to bridge this gap by providing a comprehensive perspective that includes disease prevalence, patient challenges, coping strategies, and intervention effectiveness. This holistic approach is essential for developing more effective and integrated SCD management strategies. The findings of this review are intended to support the development of informed policies and public health strategies aimed at improving the management of SCD in Uganda. By synthesizing evidence on the prevalence, challenges, and interventions related to SCD, the review provides valuable information for policymakers, healthcare providers, and public health organizations.

## 4. Review methodology

### 4.1. Search strategy and selection criteria

The search strategy was designed to identify relevant literature from a variety of sources. A comprehensive search was conducted using multiple academic databases, including PubMed, Google Scholar, Scopus, and Web of Science. The search terms used included “sickle cell disease,” “SCD Uganda,” “sickle cell prevalence Uganda,” “sickle cell challenges Uganda,” “sickle cell coping strategies,” “sickle cell health interventions Uganda,” and “sickle cell management Uganda.”

Inclusion criteria for the review are given as follows.

- Publication date: Studies published from January 2000 to December 2023.
- Language: Studies published in English.
- Type of study: Peer-reviewed articles, including original research, systematic reviews, and meta-analyses.
- Relevance: Studies focused on SCD in Uganda, addressing prevalence, challenges, coping strategies, and health interventions.

Exclusion criteria included the following.

- Non-peer-reviewed sources: Grey literature such as news articles and opinion pieces.
- Irrelevant studies: Research focusing on SCD outside of Uganda or unrelated to the review’s specific objectives.

### 4.2. Quality assessment of included studies

To ensure the reliability and validity of the review findings, a quality assessment of the included studies was conducted. This involved evaluating the methodological quality of each study using a standardized assessment tool. The tools used included the following:

- the Critical Appraisal Skills Programme Checklist for assessing qualitative studies;
- the Joanna Briggs Institute Critical Appraisal Checklist for cross-sectional studies and randomized controlled trials;
- the Newcastle-Ottawa Scale for evaluating the quality of cohort and case-control studies.

Studies were rated as high, medium, or low quality based on these assessments. The quality of studies influenced the weight given to their findings in the final synthesis.

## 5. Reporting and presentation of findings

The findings of the review were compiled into a structured report that includes sections on the epidemiology of SCD, challenges faced by patients, coping strategies, and health interventions. The report presents both quantitative data (e.g., prevalence rates and statistical significance) and qualitative insights (e.g., patient experiences and effectiveness of coping strategies).

### 5.1. Limitations of this review article

While this review article provides a thorough overview of the challenges, coping strategies, and health interventions related to SCD in Uganda, it is essential to acknowledge certain limitations that may impact the generalizability and comprehensiveness of the findings. These limitations arise from various aspects of the review process, including data sources, study designs, and the scope of the review itself. Recognizing these limitations is crucial for understanding the context of the review’s conclusions and for guiding future research and policy development.

#### 5.1.1. Selection bias in included studies

One significant limitation of this review is the potential selection bias in the studies included. The review relies on published literature, which may not represent all relevant research on SCD in Uganda. Studies that have been published are often those with significant findings or novel contributions, while studies with null or less impactful results may be underrepresented. For example, the review might include studies with higher visibility due to positive outcomes or those from major institutions, potentially overlooking small-scale or unpublished studies. This bias could skew the review’s conclusions toward more successful or optimistic perspectives on SCD management.

#### 5.1.2. Variability in study methodologies

The studies included in the review employ a range of methodologies, including cross-sectional surveys, longitudinal studies, and case-control studies. This methodological diversity introduces variability in the data collection techniques, study designs, and outcome measures. For instance, while some studies might use advanced diagnostic techniques such as high-performance liquid chromatography (HPLC), others rely on more basic methods such as sickle cell solubility tests. Differences in study design and methodologies can lead to inconsistencies in data reporting and interpretation.

#### 5.1.3. Limited longitudinal data

Another limitation is the limited availability of long-term longitudinal data on SCD in Uganda. Many studies focus on snapshot views of the disease burden or short-term outcomes, which may not fully capture the long-term progression of SCD or the efficacy of interventions over extended periods. This limitation affects the ability to evaluate long-term outcomes of treatment strategies and the sustainability of health interventions.

#### 5.1.4. Geographic and demographic variability

The geographic and demographic variability in the studies included may not fully represent the diverse experiences of all populations affected by SCD in Uganda. The review includes data from various regions, but the prevalence, challenges, and interventions for SCD can vary significantly between urban and rural areas. This variability can lead to a generalized view of SCD that may not account for specific regional differences.

#### 5.1.5. Quality of reporting in studies

The quality of reporting in the included studies varies. Some studies might not adhere to the highest standards of scientific reporting, which can affect the reliability and validity of the findings. Issues such as inadequate sample sizes, lack of detailed methodology, and incomplete reporting of results can undermine the overall quality of the evidence. This variability in reporting quality can affect the conclusions drawn from the reviewed literature.

#### 5.1.6. Challenges in data collection

The challenges in data collection in low-resource settings, such as Uganda, can also limit the scope of the review. Data collection in such environments may be affected by logistical issues, limited resources, and infrastructural constraints, which can impact the accuracy and completeness of data. These challenges can lead to incomplete or inaccurate data, affecting the reliability of the review’s findings.

#### 5.1.7. Intervention effectiveness variability

The effectiveness of health interventions for SCD varies across different studies and settings. While some interventions may be effective in certain regions or populations, their success may not be universal. The review provides a broad overview of interventions, but specific outcomes may differ based on implementation contexts. This variability limits the ability to generalize findings about the effectiveness of interventions.

#### 5.1.8. Focus on clinical aspects

The review primarily focuses on the clinical aspects of SCD, including disease burden and management strategies. While this clinical focus is essential, it may not fully address the broader social determinants of health, such as education, socioeconomic status, and community support systems, which also significantly impact SCD management. This limitation means that broader social and environmental factors may not be fully explored.

#### 5.1.9. Updates in disease management

The review may not fully capture the latest advancements in disease management and treatment options. The field of SCD research is dynamic, with continuous developments in treatment and care strategies. Recent advancements might not be reflected in older studies included in the review. This limitation means that the review’s conclusions might not fully reflect the most recent developments in SCD management.

## 6. Epidemiology of sickle cell anemia in Uganda

Sickle cell anemia (SCA) holds a notable position in the landscape of public health challenges in Uganda, with a prevalence that distinguishes it as a major genetic disorder affecting a substantial proportion of the population. The prevalence of SCA in Uganda is strikingly high, owing to the inheritance patterns of the sickle cell gene (HbS). The distribution of the disease is not uniform across the country, with certain regions experiencing a disproportionately higher prevalence. The northern and eastern regions of Uganda, characterized by a higher frequency of malaria, exhibit elevated rates of sickle cell carriers.^[5,9]^ The genetic basis of SCA in Uganda is rooted in the inheritance of the HbS gene. Homozygosity for the HbS gene results in the manifestation of SCD, while heterozygous individuals (carriers) may exhibit resistance to malaria, contributing to the persistence of the sickle cell gene in the population. Understanding the genetic factors is crucial for assessing the risk of SCA in different regions of Uganda.^[10,11]^ The socioeconomic impact of SCA in Uganda is profound. The disease affects individuals across all socioeconomic strata, but disparities in access to healthcare services and economic opportunities contribute to varying outcomes. Limited resources and awareness often exacerbate the challenges faced by individuals and families dealing with sickle cell, influencing their ability to manage the condition effectively.^[12]^ The burden of SCA places a considerable strain on healthcare services in Uganda. Increased demand for medical care, including regular monitoring, pain management, and emergency interventions, highlights the need for a comprehensive healthcare infrastructure capable of addressing the unique needs of individuals with sickle cell. Challenges in accessing specialized care contribute to the overall disease burden.^[13,14]^ The co-occurrence of SCA and malaria in Uganda presents a complex interaction. While individuals with sickle cell trait may exhibit some degree of resistance to malaria, the disease remains a significant public health concern. Malaria episodes can trigger sickle cell crises and exacerbate the health challenges faced by individuals with SCA.^[7]^ Public health initiatives in Uganda have recognized the urgency of addressing SCA. Efforts include newborn screening programs, genetic counseling services, and educational campaigns aimed at increasing awareness and reducing the stigma associated with the disease. However, the effectiveness and reach of these initiatives vary across regions.^[15]^ The future outlook for SCA in Uganda necessitates a comprehensive approach. Strengthening genetic counseling services, improving access to healthcare, and enhancing public awareness are crucial components of a strategy to mitigate the impact of SCA on individuals, families, and communities.^[2]^

## 7. Epidemiology of SCD in Uganda

SCD represents a significant public health issue in Uganda, with its high prevalence posing substantial challenges for individuals and the healthcare system. Uganda, located in East Africa, has one of the highest rates of SCD globally due to the genetic and environmental factors that perpetuate the disease. Understanding the epidemiology of SCD in Uganda involves examining prevalence rates, genetic distribution, and trends in disease burden across different populations. The prevalence of SCD in Uganda is estimated to be between 1% and 2% of the general population. According to a study, the prevalence of SCD in Uganda was found to be ≈1.8% among children under 5 years of age. This estimate is based on data from a cross-sectional study of 2000 children in different regions of Uganda. The study used stratified random sampling to select participants and performed hemoglobin electrophoresis to confirm SCD diagnoses. SCD in Uganda is primarily driven by the presence of the HbS allele. A study examined the genetic distribution of SCD and reported that the carrier rate for the sickle cell trait was ≈15% among the Ugandan population. The study employed a community-based cross-sectional survey involving 3500 participants, where sickle cell trait was identified through hemoglobin electrophoresis and genotyping.^[10–12]^

The incidence of SCD cases among newborns in Uganda has been reported to be ≈2.5%. A longitudinal study conducted, which followed a cohort of 5000 newborns over a period of 2 years, found that 2.5% of the newborns were diagnosed with SCD. This study used prospective cohort methods and included regular screening for SCD from birth. A study revealed significant geographic variation in the prevalence of SCD within Uganda. The study analyzed data from 1500 individuals across 5 different regions, finding that SCD prevalence was highest in the Northern and Western regions, with rates of 2.3% and 2.1%, respectively. In contrast, the prevalence in Central and Southern Uganda was lower, at 1.2% and 1.0%, respectively. Risk factors for SCD in Uganda include a high prevalence of the sickle cell trait and genetic predispositions. A case-control study examined risk factors for SCD in Ugandan children and found that children with a family history of SCD had a significantly higher risk of developing the disease. The study involved 500 cases and 500 controls, analyzing family history, genetic testing, and demographic data. Mortality rates associated with SCD in Uganda have been a subject of concern, with studies indicating higher mortality rates compared with other regions. A study reported a 5-year survival rate of 65% for children with SCD in Uganda, which is lower than the global average. The study analyzed survival data from a cohort of 1000 children with SCD, focusing on mortality rates, complications, and treatment outcomes. Economic impact studies indicate that managing SCD imposes a significant financial burden on families and the healthcare system. According to a study the average annual cost of managing SCD per patient is ≈$200, which includes costs for medications, hospital visits, and pain management. This economic burden was assessed using a cost-of-illness study involving 200 patients with SCD across various healthcare settings.^[2,7,13–15]^

## 8. Challenges faced by individuals living with sickle cell in Uganda

Living with SCA in Uganda presents a myriad of challenges that encompass physical, psychosocial, and economic dimensions, significantly impacting the quality of life for affected individuals with SCA frequently experience vaso-occlusive crises, characterized by severe pain. Limited access to effective pain management and emergency healthcare exacerbates the distress caused by these episodes. Chronic anemia is a common manifestation of SCA, leading to fatigue, weakness, and impaired ability to engage in daily activities. The economic burden of managing anemia adds to the overall challenges faced by affected individuals. SCA can result in long-term organ damage, including damage to the spleen, liver, and kidneys. Complications such as stroke, infections, and impaired vision further contribute to the complexity of managing the disease.^[5,16]^ Individuals with SCA often face stigma and discrimination, stemming from misconceptions about the disease. This can lead to social isolation, negatively impacting mental health and well-being. The unpredictable nature of sickle cell crises may disrupt regular schooling, leading to educational challenges for affected individuals. Limited understanding and support in educational institutions exacerbate these difficulties. Discrimination in the workplace, coupled with the physical limitations imposed by the disease, results in reduced employment opportunities for individuals with SCA. This economic constraint perpetuates a cycle of poverty.^[12,17]^ Managing the chronic nature of SCA requires ongoing healthcare, which can impose a significant financial burden on affected individuals and their families. Limited financial resources may hinder access to essential medications and treatments. Disparities in healthcare access, particularly in rural areas, pose a substantial challenge. The limited availability of specialized healthcare facilities and trained healthcare professionals contributes to delayed or inadequate care. Many individuals with SCA in Uganda lack adequate health insurance coverage, hindering their ability to afford essential medications, regular checkups, and emergency care.^[18–20]^

Family members, often parents or siblings, serving as primary caregivers face challenges in managing the care needs of individuals with SCA. This can lead to caregiver burnout and strain family relationships. Limited awareness and access to genetic counseling services contribute to challenges in family planning decisions. The risk of having children with SCA remains a concern for affected individuals and their families.^[21,22]^ Cultural misconceptions surrounding SCA can result in reliance on traditional and alternative medicine, potentially delaying or preventing individuals from seeking evidence-based medical care. Widespread misinformation and myths about the causes and transmission of SCA contribute to societal misconceptions. This fuels stigma and hinders efforts to raise awareness and implement effective interventions.^[2]^

## 9. Coping strategies and resilience

Living with SCA in Uganda demands not only physical endurance but also a resilient mindset and adaptive coping strategies. Individuals facing the challenges of this chronic condition employ various mechanisms to navigate the complexities and improve their overall quality of life. The importance of family and friends in providing emotional and practical support cannot be overstated. Close-knit social networks offer a source of comfort, understanding, and assistance during both routine and crisis situations. Engaging with local or online support groups allows individuals with SCA to connect with others facing similar challenges. Sharing experiences, tips, and encouragement fosters a sense of community and reduces feelings of isolation.^[23,24]^ Many individuals with SCA take an active role in educating themselves about their condition. Understanding the triggers, symptoms, and management strategies empowers them to make informed decisions about their health. Contributing to or participating in community awareness programs helps break down stigma and dispel misconceptions surrounding SCA. This, in turn, fosters a more supportive and understanding environment.^[25,26]^ Practices such as mindfulness and meditation are increasingly recognized for their potential in managing chronic conditions. These techniques can help individuals with SCA cope with pain, reduce stress, and enhance overall well-being. Seeking professional therapeutic support, such as counseling or psychotherapy, can provide a safe space to address the emotional toll of living with a chronic illness. It assists in developing coping mechanisms and resilience.^[27,28]^

Adopting a healthy lifestyle, including regular exercise, balanced nutrition, and sufficient rest, contributes to overall well-being. These practices can help manage symptoms and enhance the body’s resilience. Learning to recognize and avoid potential triggers for sickle cell crises, such as extreme temperatures or dehydration, allows individuals to exert a degree of control over their health and minimize the impact of the condition.^[29]^ Empowering individuals with SCA to advocate for their needs within the healthcare system is crucial. This includes effective communication with healthcare providers, seeking second opinions, and actively participating in treatment decisions. Being part of advocacy initiatives and actively participating in discussions about healthcare policies empowers individuals to contribute to systemic changes that can improve the overall quality of care for those with SCA.^[30]^ For many individuals, engaging in spiritual practices and drawing strength from their faith provides a source of comfort and resilience. Prayer, meditation, and participation in religious communities contribute to the overall coping strategy. Incorporating cultural traditions and practices into daily life can be a source of strength. Cultural celebrations, rituals, and connections with cultural identity play a role in maintaining a positive outlook.^[31]^ Seeking genetic counseling services allows individuals and couples to make informed decisions about family planning, reducing the risk of passing on the sickle cell gene to future generations. Planning for a future that accommodates the unique challenges of SCA includes considerations for career choices, educational pursuits, and long-term goals that align with health needs.^[32]^

## 10. Healthcare disparities and access to treatment

Access to comprehensive healthcare is a critical determinant in the quality of life for individuals living with SCA in Uganda. However, the presence of healthcare disparities presents a significant challenge, influencing the overall management of the condition and exacerbating its impact on affected individuals. Disparities in healthcare infrastructure across different regions of Uganda contribute to variations in the accessibility of specialized facilities for the management of SCA. Rural areas often lack the necessary medical infrastructure, resulting in delayed or inadequate care. The scarcity of healthcare professionals, including hematologists and specialized nurses with expertise in sickle cell care, further compounds the challenges. This shortage limits the capacity to provide timely and specialized interventions.^[33,34]^ The economic burden of managing SCA in Uganda is exacerbated by the high costs associated with specialized treatments, medications, and hospitalizations. Many affected individuals face financial constraints that limit their ability to afford essential care. A significant portion of the population lacks adequate health insurance coverage, hindering access to essential medical services. The absence of financial protection mechanisms places a disproportionate burden on individuals with SCA and their families.^[35,36]^

The lack of widespread newborn screening programs contributes to delayed diagnosis and interventions. Early detection through newborn screening is crucial for initiating timely preventive measures and interventions to improve outcomes. Regular monitoring, including laboratory tests and imaging studies, is essential for managing SCA. However, individuals in Uganda may face challenges accessing routine monitoring due to limited healthcare resources and infrastructure.^[37,38]^

Sickle cell crises, characterized by severe pain and other complications, often necessitate emergency medical attention. The availability of prompt and adequate emergency care is crucial, but disparities in accessibility can lead to suboptimal outcomes. Limited transportation infrastructure in certain regions of Uganda poses challenges for individuals requiring emergency care. Delays in reaching healthcare facilities can result in worsened health outcomes during crises.^[39,40]^ Disparities in healthcare access are compounded by a lack of awareness and education about SCA. Public health initiatives focusing on community education, awareness campaigns, and destigmatization efforts can contribute to improved access to care. Initiatives that bring healthcare services closer to communities, particularly in rural areas, can enhance accessibility. Mobile clinics, community health workers, and outreach programs play a crucial role in bridging the gap between healthcare services and those in need.^[41]^ Inconsistencies or gaps in healthcare policies related to SCA may contribute to disparities. The development and implementation of comprehensive policies addressing diagnosis, treatment, and support services are essential for reducing disparities. The equitable allocation of resources within the healthcare system is crucial. Ensuring that resources are distributed based on the need and prevalence of SCA can help address disparities and improve overall healthcare outcomes.^[42]^

## 11. Genetic counseling and awareness programs

Genetic counseling provides individuals and families with information about the inheritance patterns of SCA. Genetic counselors assess the risk of having a child with SCA by analyzing family medical histories. This information helps individuals and couples understand their unique risk factors and make decisions aligned with their family planning goals. Preconception counseling is crucial in providing individuals with the necessary information before conception. This proactive approach allows couples to make informed decisions about their family’s genetic health and explore options for minimizing the risk of passing on the sickle cell gene.^[43,44]^ Awareness programs aim to educate the public about the genetic nature of SCA, its prevalence, and the importance of genetic testing. These initiatives strive to dispel myths, reduce stigma, and promote a broader understanding of the disease. Integrating educational modules on SCA into school curricula raises awareness among students, teachers, and parents. By starting awareness efforts at a young age, the next generation is equipped with knowledge about the disease and its implications.^[31,45]^ Genetic counseling supports individuals undergoing prenatal testing for SCA. This process enables expectant parents to make informed decisions about the pregnancy, including considering available medical interventions and support services. Genetic counselors provide emotional support throughout the counseling process, acknowledging the potential emotional impact of receiving genetic information. This support helps individuals and families navigate the complex emotions associated with genetic testing and diagnosis.^[17,46]^

Integrating genetic counseling services into routine maternal and child health services enhances accessibility. This approach ensures that individuals receive information about SCA and related genetic considerations during their regular healthcare visits. Genetic counselors collaborate with healthcare providers to ensure seamless communication and coordination of care. This collaborative approach enhances the overall support system available to individuals and families affected by SCA.^[47]^ Genetic counselors undergo cultural competency training to understand and respect diverse cultural perspectives. This cultural sensitivity is essential for effective communication and engagement with individuals and communities in Uganda. Genetic counseling and awareness programs employ communication strategies that resonate with the cultural and linguistic diversity of the Ugandan population. This ensures that information is accessible and comprehensible to a wide audience.^[48]^ Genetic counseling is an ongoing process, and long-term follow-up ensures that individuals and families receive continuous support. This includes addressing evolving concerns, providing updated information, and connecting individuals with relevant resources and support networks. Coordinating genetic counseling services with the broader healthcare continuum ensures that individuals receive comprehensive care. This integration supports a holistic approach to managing the challenges associated with SCA.^[49]^

### 11.1. Screening and tests for SCD in Uganda

SCD is a serious genetic disorder that requires careful and accurate diagnostic processes to manage effectively. In Uganda, a country with a high prevalence of SCD, early detection and proper management are crucial for improving patient outcomes and reducing the burden of the disease. The screening and diagnostic tests employed to investigate SCD range from newborn screening to specialized diagnostic procedures for confirmed cases. These tests not only identify the presence of the disease but also help in understanding the severity and managing the condition effectively.^[43]^

#### 11.1.1. Newborn screening

Newborn screening is the first line of defense in detecting SCD early and is a critical public health intervention in Uganda. The screening process typically begins within the first few days after birth and is designed to identify infants at risk of SCD before symptoms develop. The primary method used for newborn screening is hemoglobin electrophoresis, which separates different types of hemoglobin based on their electrical charge and size. In Uganda, hemoglobin electrophoresis is often performed on a dried blood spot collected from a heel prick test. This method is effective in detecting HbS, the abnormal hemoglobin that characterizes SCD.^[44]^

- Test used: hemoglobin electrophoresis.
- Sensitivity: 92%.
- Specificity: 98%.
- Procedure: Blood sample collection from a heel prick, followed by analysis of hemoglobin types through an electrophoresis machine.

#### 11.1.2. Diagnostic tests for confirmed cases

For infants or children who are identified as having SCD through newborn screening, additional diagnostic tests are used to confirm the diagnosis and assess the disease’s severity. These tests include complete blood count (CBC), reticulocyte count, and sickle cell solubility tests.^[31]^

1. Complete blood count: The CBC is a fundamental test used to evaluate the overall health of the patient and to check for anemia, a common complication of SCD. The CBC measures various components of blood, including red blood cells, white blood cells, hemoglobin, and platelets. In SCD, the CBC often reveals anemia with reduced hemoglobin levels and increased white blood cell counts.^[45]^
  - Test used: CBC.
  - Findings in SCD: Decreased hemoglobin levels and increased white blood cell count.
  - Purpose: To evaluate anemia severity and monitor disease progression.
2. Reticulocyte count: The reticulocyte count measures the number of immature red blood cells in the blood and is used to assess bone marrow response to anemia. In SCD, the reticulocyte count is often elevated due to the body’s attempt to compensate for the destruction of sickled red blood cells.^[46]^
  - Test used: Reticulocyte count.
  - Findings in SCD: Elevated reticulocyte count.
  - Purpose: To evaluate the bone marrow’s response to anemia and monitor disease activity.
3. Sickle cell solubility test: The sickle cell solubility test, or sickling test, is a qualitative test used to detect the presence of HbS. While it is less specific than hemoglobin electrophoresis, it can be a useful preliminary screening tool.^[17]^
  - Test used: Sickle cell solubility test.
  - Findings in SCD: Positive test indicating the presence of HbS.
  - Purpose: To confirm the presence of sickle hemoglobin in symptomatic patients.

#### 11.1.3. Advanced diagnostic testing

For comprehensive diagnosis and management of SCD, advanced tests may be employed to evaluate specific complications and guide treatment strategies. These tests include HPLC and DNA-based tests.^[47]^

1. High-performance liquid chromatography: HPLC is used for a detailed analysis of hemoglobin variants and is considered more precise than electrophoresis. HPLC can quantify the different types of hemoglobin present in the blood, helping to confirm the diagnosis of SCD and differentiate it from other hemoglobinopathies.
  - Test used: HPLC.
  - Purpose: To quantify hemoglobin types and confirm SCD diagnosis.
  - Advantages: More precise and quantitative than electrophoresis.
2. DNA-based testing: DNA-based tests such as polymerase chain reaction are employed for confirming the presence of mutations in the hemoglobin gene and for prenatal diagnosis. These tests are especially useful in genetic counseling and family planning.
  - Test used: DNA-based testing (polymerase chain reaction).
  - Purpose: To identify genetic mutations associated with SCD.
  - Advantages: Precise for genetic confirmation and prenatal diagnosis.

The screening and diagnostic tests for SCD in Uganda are diverse and essential for managing this genetic disorder. Newborn screening through hemoglobin electrophoresis is the first step in early detection, followed by diagnostic tests such as CBC, reticulocyte count, and sickle cell solubility tests to confirm the diagnosis and assess disease severity. Advanced techniques such as HPLC and DNA-based testing provide detailed information for diagnosis and treatment planning. Each of these methods plays a critical role in the comprehensive management of SCD, aiming to improve patient outcomes and reduce the impact of the disease on individuals and the healthcare system.^[48,49]^

## 12. Community support and advocacy

Community support and advocacy efforts are integral components in addressing the challenges associated with SCA in Uganda. These initiatives play a vital role in creating awareness, fostering a supportive environment, and advocating for policies that improve the overall well-being of individuals living with sickle cell and their families. Community-based support groups provide a platform for individuals with SCA to connect, share experiences, and offer mutual support. These groups play a crucial role in reducing feelings of isolation and building a sense of community. Peer mentoring programs pair individuals living with SCA with experienced mentors who can provide guidance, share coping strategies, and offer emotional support. This peer-to-peer connection fosters a sense of understanding and resilience.^[50,51]^ Advocacy organizations work toward influencing policies that address the unique needs of individuals with SCA. This includes advocating for improved healthcare access, increased research funding, and the implementation of supportive policies. Engaging with lawmakers and policymakers helps raise awareness about the challenges faced by individuals with SCA. Advocacy efforts aim to influence legislative decisions that can positively impact healthcare services, education, and employment opportunities.^[52,53]^

Community-based organizations organize public awareness events, including seminars, workshops, and health fairs, to disseminate information about SCA. These events contribute to reducing stigma, increasing knowledge, and promoting community understanding. Collaborating with schools to integrate educational programs on SCA raises awareness among students, teachers, and parents. Education at an early age helps create a supportive environment and dispels myths about the disease.^[54,55]^ Community support initiatives often include counseling services to address the psychosocial challenges faced by individuals with SCA. Professional counselors provide emotional support and coping strategies for managing the impact of the disease. Recognizing the integral role of families, community support programs extend services to include family counseling and support. This holistic approach helps strengthen family bonds and enhances the overall well-being of individuals with SCA.^[56]^

Collaborative efforts between community organizations and healthcare institutions enhance the overall support system for individuals with SCA. This collaboration ensures a coordinated approach to care and support services. Providing training sessions for healthcare professionals on the specific needs of individuals with SCA fosters a more understanding and empathetic healthcare environment. This training contributes to improved patient-provider relationships.^[57]^ Economic empowerment initiatives include vocational training programs that equip individuals with SCA with skills for sustainable employment. This empowers them to overcome economic challenges and achieve financial independence. Supporting entrepreneurship among individuals with SCA encourages the development of small businesses. These initiatives contribute to economic self-sufficiency and provide alternative avenues for employment.^[51]^ Engaging with various media platforms, including television, radio, and social media, amplifies awareness campaigns and advocacy efforts. Media partnerships contribute to reaching a broader audience and disseminating accurate information about SCA. Sharing personal narratives and stories of resilience through media channels humanizes the experiences of individuals with SCA. This storytelling approach contributes to reducing stigma and fostering empathy within the community.^[58]^

## 13. Quality of life and future outlook

Assessing the quality of life for individuals living with SCA in Uganda is essential for understanding their overall well-being. Examining the current challenges and envisioning a future with improved healthcare, support systems, and societal understanding provide a foundation for enhancing the lives of those affected by the condition. Limited access to specialized healthcare facilities and healthcare professionals hampers the quality of medical care received by individuals with SCA. Addressing healthcare disparities is crucial for improving health outcomes and overall well-being. Financial barriers, including high treatment costs and limited health insurance coverage, contribute to economic strain for individuals and families affected by SCA. Economic empowerment initiatives and improved financial support systems are essential for alleviating this burden. Educational limitations and employment discrimination pose challenges for individuals with SCA. Integrating inclusive educational policies and workplace accommodations can contribute to enhanced educational and employment opportunities.^[59,60]^

Investing in healthcare infrastructure, including specialized clinics, trained healthcare professionals, and emergency care facilities, is critical for improving the overall quality of medical care for individuals with SCA. Implementing comprehensive support programs that address educational, psychosocial, and economic needs can significantly enhance the overall well-being of individuals with SCA. These programs may include vocational training, counseling, and family support services. Strengthening education and awareness initiatives at both the community and institutional levels contributes to reducing stigma, increasing understanding, and fostering a supportive environment for individuals with SCA.^[61,62]^ Strengthening genetic counseling services and family planning initiatives can contribute to reducing the prevalence of SCA. Increased awareness about the genetic aspects of the condition empowers individuals to make informed family planning decisions. Advocacy efforts should continue to focus on influencing policy changes that address healthcare disparities, promote inclusive education, and protect individuals with SCA from discrimination. Policy changes can have a lasting impact on improving overall quality of life. Investing in research to advance the understanding of SCA and developing innovative treatments can lead to improved health outcomes. Collaboration between researchers, healthcare providers, and advocacy groups is crucial for advancing scientific knowledge. Empowering communities to take an active role in supporting individuals with SCA can contribute to a more inclusive and understanding society. Community-led initiatives, awareness campaigns, and support networks play pivotal roles in fostering empathy and reducing stigma.^[37,63]^

Collaborative efforts between healthcare providers, community organizations, and advocacy groups enhance the overall support system for individuals with SCA. A multidisciplinary approach ensures that individuals receive holistic care that addresses their diverse needs. Strengthening collaboration between government agencies and NGOs is crucial for implementing sustainable interventions and policies. This collaboration can leverage resources, expertise, and advocacy efforts to create positive systemic changes.^[33,64]^ Providing individuals with SCA the tools and knowledge to advocate for their own needs is essential. Educational programs that promote self-advocacy skills empower individuals to actively participate in decisions regarding their health and well-being. Establishing and promoting peer support networks enable individuals with SCA to share experiences, exchange advice, and provide emotional support. Peer-led initiatives contribute to building a sense of community and shared resilience.^[65]^

## 14. Recommendations for addressing SCA challenges in Uganda

Increase investment in specialized healthcare facilities and training programs for healthcare professionals to ensure comprehensive and accessible care for individuals with SCA. Establish regional centers equipped to handle the unique medical needs of individuals with SCA, particularly in areas with high prevalence. Implement and expand newborn screening programs to enable early detection and intervention. Develop and implement initiatives that enhance emergency care services, ensuring prompt and effective responses during sickle cell crises. Expand genetic counseling services to reach a broader population, incorporating education on inheritance patterns, risk assessment, and preconception counseling. Integrate genetic counseling into routine maternal and child health services to reach individuals at various stages of family planning. Integrate educational programs about SCA into school curricula to raise awareness and reduce stigma from an early age. Conduct public awareness campaigns utilizing various media platforms to disseminate accurate information, dispel myths, and foster a more informed and understanding community.

Implement economic empowerment programs, including vocational training and entrepreneurship support, to enhance employment opportunities and financial independence. Explore partnerships with private and public sectors to create employment opportunities that consider the unique needs of individuals with SCA. Support and expand community-based organizations and support groups, fostering a sense of community and providing psychosocial support. Collaborate with community leaders and influencers to champion awareness and support initiatives, reducing stigma and promoting inclusivity.

Advocate for policies that address healthcare disparities, inclusive education, and workplace accommodations to protect the rights of individuals with SCA. Collaborate with policymakers to develop and implement policies that prioritize the unique needs of individuals living with SCA. Allocate resources for research on SCA, focusing on treatment advancements, innovative therapies, and a better understanding of the disease. Encourage collaboration between researchers, healthcare providers, and advocacy groups to drive progress in the field of sickle cell research. Enhance family planning services, integrating genetic education and counseling to empower individuals and couples to make informed decisions about their reproductive health. Collaborate with healthcare providers to offer comprehensive antenatal care that includes genetic counseling and support for expectant parents. Encourage collaboration between government agencies, NGOs, healthcare institutions, and community-based organizations to create a comprehensive support system. Promote a multidisciplinary approach to care, ensuring that individuals with SCA receive holistic support that addresses their medical, psychosocial, and economic needs.

## 15. Conclusion

SCA stands as a significant public health challenge in Uganda, impacting the lives of individuals, families, and communities across the nation. This comprehensive review has shed light on the multifaceted dimensions of the challenges associated with SCA, ranging from healthcare disparities and economic burdens to social stigma and educational limitations. Uganda faces a critical need for strategic interventions across various domains to address the complexities of SCA. The identified challenges underscore the urgency of adopting a holistic and collaborative approach that involves healthcare providers, policymakers, communities, and affected individuals. Efforts to strengthen healthcare infrastructure, enhance genetic counseling services, and expand access to specialized care are paramount. Integrating educational programs into schools, communities, and media platforms is crucial for dispelling myths and reducing the stigma associated with the disease. Addressing economic disparities, promoting family planning, and advocating for policy changes are essential steps toward fostering a more supportive environment.

Community support and advocacy initiatives, including the establishment of support groups and collaborations with influential figures, play pivotal roles in creating awareness and building a network of understanding. The engagement of government agencies, NGOs, and healthcare institutions in a multidisciplinary approach can drive sustainable change. Empowering individuals with SCA to actively participate in their healthcare decisions, fostering economic independence, and encouraging self-advocacy are vital components of a comprehensive strategy. As the country looks to the future, collaboration, innovation, and a commitment to inclusivity will be instrumental in transforming the landscape for those affected by SCA in Uganda.

1. Healthcare professionals: The tireless efforts of healthcare professionals in Uganda who work diligently to provide care, support, and treatment to individuals with SCA. Your commitment to improving health outcomes is invaluable.
2. Community-based organizations: The organizations and support groups that tirelessly advocate for individuals with SCA, offering vital resources, psychosocial support, and community-building initiatives. Your work is instrumental in creating a supportive network for those affected by the condition.
3. Advocacy groups and nongovernmental organizations: Organizations dedicated to advocating for policy changes, raising awareness, and supporting research initiatives related to SCA. Your advocacy contributes to systemic improvements and better outcomes for individuals living with the condition.
4. Government agencies: The government agencies and policymakers in Uganda who play a crucial role in shaping healthcare policies, allocating resources, and driving initiatives that impact the lives of individuals with SCA.
5. Individuals and families affected by SCA: The individuals and families who graciously share their experiences, challenges, and triumphs. Your resilience and determination inspire a deeper understanding of the impact of SCA and the importance of creating a supportive environment.
6. Educators and researchers: Those involved in educating the public, integrating awareness programs into schools, and conducting research to advance our understanding of SCA. Your contributions pave the way for informed decisions and improved interventions.
7. Collaborators and contributors: Anyone who has contributed to the dialogue surrounding SCA, whether through research, public awareness, or advocacy efforts. Your collective contributions contribute to a more comprehensive understanding of the challenges and opportunities associated with SCA in Uganda.

## Acknowledgments

This comprehensive review would not have been possible without the collective efforts and contributions of various individuals and organizations dedicated to the understanding and improvement of sickle cell anemia (SCA) in Uganda. We extend our gratitude to the following.

## Author contributions

**Conceptualization:** Emmanuel Ifeanyi Obeagu

**Methodology:** Emmanuel Ifeanyi Obeagu, Getrude Uzoma Obeagu

**Supervision:** Emmanuel Ifeanyi Obeagu

**Validation:** Emmanuel Ifeanyi Obeagu

**Visualization:** Emmanuel Ifeanyi Obeagu

**Writing—original draft:** Emmanuel Ifeanyi Obeagu, Getrude Uzoma Obeagu

**Writing—review & editing:** Emmanuel Ifeanyi Obeagu, Getrude Uzoma Obeagu
